# ReCoil - an algorithm for compression of extremely large datasets of dna data

**DOI:** 10.1186/1748-7188-6-23

**Published:** 2011-10-11

**Authors:** Vladimir Yanovsky

**Affiliations:** 1 Department of Computer Science, University of Toronto, Toronto, Canada

## Abstract

The growing volume of generated DNA sequencing data makes the problem of its long term storage increasingly important. In this work we present ReCoil - an I/O efficient external memory algorithm designed for compression of very large collections of short reads DNA data. Typically each position of DNA sequence is covered by multiple reads of a short read dataset and our algorithm makes use of resulting redundancy to achieve high compression rate.

While compression based on encoding mismatches between the dataset and a similar reference can yield high compression rate, good quality reference sequence may be unavailable. Instead, ReCoil's compression is based on encoding the differences between similar or overlapping reads. As such reads may appear at large distances from each other in the dataset and since random access memory is a limited resource, ReCoil is designed to work efficiently in external memory, leveraging high bandwidth of modern hard disk drives.

## 1 Introduction

### 1.1 Motivation

High speeds and relatively low prices of High Throughput Sequencing (HTS) technologies led to their widespread use for various kinds of applications, making it important to store high volumes of generated sequencing data efficiently.

Given a genetic sequence, an HTS sequencer outputs a set of subsequences, called *reads*, of the sequence. Unlike the more expensive sequencing technologies that were used, for example, for the Human Genome Project, the HTS reads usually have higher error rate and shorter lengths. On the other hand, the datasets produced by an HTS sequencer are usually of high *coverage*, i.e. they can have many different reads overlapping at each position, making them highly compressible. In this work we address the problem of compression of datasets of HTS reads.

Previous research [1] show that for the sequence of a human genome it is hard to achieve compression rate significantly better than a trivial two bits per nucleotide. Hence the algorithms for compression of HTS datasets must take advantage of the self-similarity due to read overlaps. One difficulty that must be overcome is that for huge HTS datasets similar or overlapping reads can be at great distance from each other in the input and splitting it into smaller chunks will miss these similarities. Hence our goal was a compression algorithm that works on the whole dataset at once, using external memory without a significant hit in performance.

### 1.2 Previous Work

#### DNA Sequence Compression

DNA sequence contains a large number of approximate repeats. Yet, general purpose compression tools, such as *gzip *or *bzip2*, cannot make use of this redundancy in order to achieve compression rate for DNA sequences or datasets significantly better than the trivial encoding of two bits for each of four possible nucleotides [1].

Specialized DNA compression algorithms find approximate repeats in the sequence and then attempt to encode efficiently the differences between the instances of the repeat. The best compression to date for a single sequence is achieved by DNACompress [1]. This tool is based on PatternHunter [2] - a package for sequence homology search similar to BLAST. DNACompress runs PatternHunter to find approximate repeats in the sequence, then sorts them such that long high similarity repeats appear first. During the encoding stage DNACompress extracts the highest scoring approximate repeat and encodes all its instances using edit operations transforming between them. Then the list of all hits reported by PatternHunter is filtered out of all sequences overlapping with those encoded by the step. This step is repeated until the remaining highest scoring hit has the score below some threshold. While it is possible to modify DNACompress for the compression of the datasets of HTS reads, it is not designed to handle large input size: in [3] the authors tested DNACompress and found it could not handle even the smallest of their datasets.

#### Genomic Dataset Compressed Indexing

Several works consider the problem of compressed full-text self-indexing of a DNA dataset. For example, Makinen et al. [4] describes an algorithm to build a compressed *self-index *for a collection of strings of equal length generated by the SNPs in a single string. The index they introduce is a suffix array based data structure that given a string *T *permits the following operations:

**- ***Count*(*T*) - counts the number of appearances of *T *as a substring of the strings in the collection

**- ***Search*(*T*) - outputs the positions where *T *appears as a substring in the strings of the collection

**- ***Display*(*i*, *j*, *k*) - displays *S_k_*[*i *⋯ *j*], where *S_k _*is the *k*'th string of the collection

While compressed full text indices achieve lower compression rate than known dedicated compression utilities [5], they address a different set of tradeoffs than our work, in which we attempt to achieve the best compression rate.

#### Compression Using Known Reference Sequence

Knowing a reference genome makes it possible to achieve very high compression rate by storing only the differences between the sequences. In [6] this approach was used for compression of a single human genome. To further improve the compression rate, the algorithm stores the mutations in the target genome using public databases of known genomic variations. The result was just a 4 MB compressed file.

Other tools, such as SlimGene [7] and MZip [8], address the problem of compressing a dataset by mapping each read against a highly similar reference sequence and storing only the differences between the read and the reference. These tools receive as an input alignments of each read produced by a mapping program and use methods for variable length integer coding, such as Huffman coding, to efficiently store the alignments. In addition to compressing DNA sequences, SlimGene [7] and MZip [8] discuss ways to efficiently store the quality scores - the values attached to each position in the read, reflecting the confidence level of the sequencer in its output for that position. Due to relatively high space requirement of the quality scores, the works suggest to store them using lossy compression, arguing that, in practice, storing exact values would not be beneficial in most applications. In this work we only address compression of DNA sequences.

There are limitations to the reference-based approach. The reference sequence might not be available, for example for a metagenomic dataset, or not be similar enough for organisms with very high polymorphism. Also, there is a strong practical advantage of compression methods that keep all the data required for the decompression in the compressed file. Decoding a reference-based encoded sequence, especially following a transfer or long term storage, can be inconvenient, as the decoder must be able to access the reference, likely to be stored separately from the compressed dataset due to its size.

Reconstruction of the original (or assembled) sequence, if it is not given, and using it as the reference for storing the dataset is not a viable option: the problem of genome assembly, especially in the presence of sequencing errors, is computationally too expensive and there are no known *De Novo *assembly algorithms that would work on a workstation [9]. Hence our interest in this work to consider the problem of compression of a dataset under assumption that the original sequence is unknown.

#### Coil

[3] is a tool designed for compression of datasets of DNA sequences. Coil builds a hash table *H *of the lists of locations of all DNA strings of length *k*, called *k*-mers, and uses it to find the subset of sequences similar to each sequence in the input dataset *S*. This subset can be found as follows: for a sequence *S_i _*compute the set of all *k*-mers in *S_i _*and use hash table *H *to merge the lists of sequences containing them. Those sequences that appear frequently enough in the merged list are likely to be similar to *Si*. Since this merged list has to be found for each input sequence, in order to speed up execution, Coil uses the following Least Recently Used (*LRU *) heuristic for *k*-mers' list merging. When Coil computes the set of sequences similar to *S_i_*, instead of merging all location lists for all *k*-mers in *S_i _*into one possibly very long list, it manages only a fixed number *r *of small, fixed-size LRU arrays *A_j_*, 0 ≤ *j < r*, which together hold the most recently accessed sequences that share a *k*-mer with *S_i_*.

The sequences in each *A_j _*are maintained in the order of time since their last sighting and for each sequence Coil counts the number of times the sequence was accessed since it was placed in *A_j _*last time. Scanning through the lists of sequences containing each *k*-mer in *S_i_*, each such sequence *s *is merged into *A_smodr _*as follows: if *s *is already present, its counter is incremented and the sequence is moved to the front of the array; otherwise all sequences in the array are shifted back to make space for *s*, discarding the least recently seen sequence if the array was already full. The size of this array is necessarily small, otherwise the updates to it would be too expensive. This may result in sequences being placed in and removed from some *A_j _*repeatedly in the cases when *k*-mer lists are expected to be long such as for smaller values of *k *and for datasets containing many short reads, like those produced by HTS.

In the next stage Coil builds a weighted *similarity graph *with the vertices corresponding to the sequences of the dataset and an edge between a sequence *S_i _*and each sequence in each array *A_j_*. The weight of each edge equals the corresponding appearance counter.

In the encoding step, in a manner reminiscent of phylogenetic tree approximations, Coil uses the Maximum Spanning Tree in the similarity graph as its encoding tree. Coil stores the read in the root of the spanning tree explicitly and then encodes, in the order of a preorder traversal of the tree, each child with the set of differences between it and its parent. If the weight of the edit script is above some threshold, the child is stored explicitly. Finally, Coil compresses the scripts with a general purpose compression utility such as *bzip2*.

### 1.3 Our Contribution

In this work we design a compression algorithm suitable for very large datasets. As for this task the internal memory is the main bottleneck, ReCoil does not assume that the input or the data structures created by the algorithms fit in RAM. Instead, ReCoil uses hard disks as its working memory. While bandwidth of modern disks is comparable to that of internal memory, their access times are many orders of magnitude slower. Hence, if the algorithm is designed in a way to minimize the number of random accesses, its performance can become competitive to the RAM-based algorithms. All steps of ReCoil were designed as reductions to either scanning or sorting of the input - two tasks that can be done I/O-efficiently. Decompression can be reduced to input scanning, hence it is very fast.

The main contribution of the ReCoil algorithm is overcoming the necessity to split large datasets into smaller chunks in order to fit in Random Access Memory. In addition, while sharing the idea of spanning tree based compression with Coil, our algorithm improves over Coil in various ways:

**- **ReCoil makes use of highly repetitive *k*-mers. Coil does not compress high count repeats, storing them explicitly.

**- **ReCoil's encoding scheme allows for encoding of matches of arbitrary length, while the only edit operations allowed by Coil are insertions, deletion and substitution of a single nucleotide. While SNPs are the most frequent mutation, Coil's encoding scheme is not efficient for encoding of the similarity due to overlaps between reads, which are the main source of compressibility of HTS datasets.

**- **ReCoil uses effectively the complementarity of the DNA in the dataset.

We compare our algorithm to the general purpose compressors such as *bzip2 *and *7-zip *as well as to Coil. It should be noted that one of the reasons ReCoil and Coil can achieve better compression than the general purpose tools is that the order of reads in the dataset is not significant and they can safely disregard it. The source code is available from the author upon request.

## 2 Methods

ReCoil uses the natural idea that if two strings *s*_1 _and *s*_2 _are sufficiently similar, then it is more space efficient to store one of the strings and the differences between it and the second string, then to store both strings explicitly.

### 2.1 Memory Model and Basic Definitions

#### Memory Model

We use the standard model of Aggarwal and Vitter [10] for analysis of external memory algorithms. In this model the machine has Random Access Memory (RAM) of size *M *and a disk of unlimited size. The CPU can operate only on data that is currently in RAM. Data is transferred between RAM and the disk in blocks of *B *consecutive elements, where *B < M*. In this model performance of algorithms is measured in terms of number of accesses to the disk, reflecting an observation that runtimes of most disk-bound algorithms are dominated by disk accesses. Aggarwal and Vitter [10] prove that in this model scanning an array of size *n *has complexity *Θ *(*Scan n*) = *Θ*(*n/B*) and sorting requires $\Theta \left(Sort\;n\right)=\Theta \left(\frac{n}{B}\log_{\frac{M}{B}}\frac{n}{B}\right)$ accesses to the disk. It is common to express I/O performance of an algorithm on data of size *n *in terms of the *Sort *and the *Scan *complexity.

#### Basic Definitions

Before proceeding to the description of the algorithm let us introduce some basic definitions:

**- **For an integer *k *and a string *s *we call the set of all substrings of *s *of length *k *the *seeds *or *k*-mers contained in *s*.

**- **Maximal exact matches (MEMs) are exact matches between two strings that cannot be extended in either direction towards the beginning or end of two strings without allowing for a mismatch.

Next we will give a brief overview of the compression algorithm and explain it in more detail later.

### 2.2 Overview

To encode the similarities between two reads ReCoil uses the set of MEMs between them: in order to encode *s*_2 _given *s*_1 _we store the locations of MEMs shared by *s*_1 _and *s*_2_, and the letters of *s*_2 _not in these MEMs.

The similarity graph for the dataset is defined as a weighted undirected graph with vertices corresponding to the reads of the dataset. For any two reads *s*_1 _and *s*_2 _the weight of the edge connecting them should reflect the profitability of storing *s*_1 _and the differences between it and *s*_2 _versus storing both reads explicitly.

The encoding algorithm has four major steps, all of which have at most *O*(*Sort n*) I/O complexity:

**- **Compute the similarity graph.

**- **Find the set of encoding edges - the edges of a maximum spanning tree (MST) in the similarity graph (there may in general be multiple MSTs).

**- **Pick the root of the MST arbitrarily and store the sequence in the root explicitly.

**- **Traverse the MST, encoding each node using the set of the Maximum Exact Matches (MEMs) between the node's read and the read of its parent in the MST.

Note that while the similarity graph is defined here as a clique, unprofitable edges of high weight are not used in the encoding and may be excluded from the graph. In addition, for simplicity of the MST construction we make the graph connected by adding for each *i *an edge of high weight between reads *r_i _*and *r_i_*_+1_.

### 2.3 Construction of the Similarity Graph

**Figure 1:**
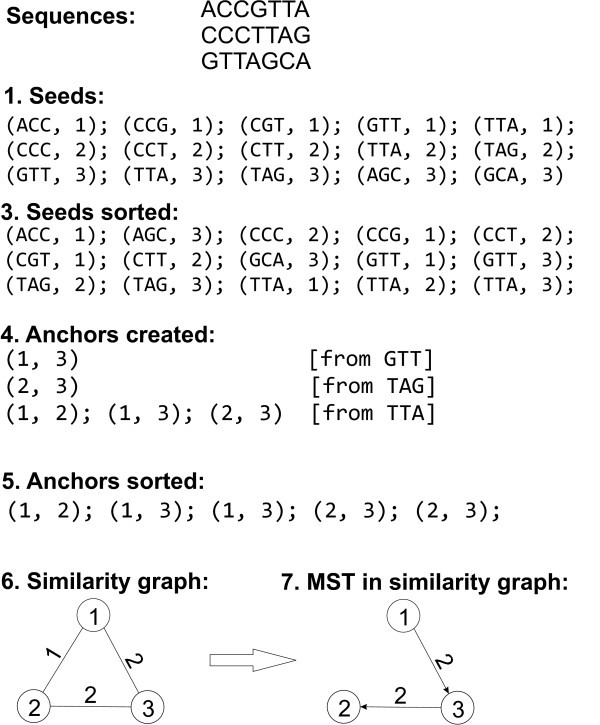
**Top level illustration of the algorithm of Section 2.3 for construction of the *similarity graph *for three sequences**. To simplify the illustration we do not consider here the reverse complement reads and the filtering of Step 2. The encoding that corresponds to the tree can be found in Section 2.4.

We define the weight of an edge of the *similarity graph *to be equal to the number of *k*-mers shared by the reads corresponding to the edge's endpoints. As for large datasets this graph cannot be held in memory, its construction was reduced to an external memory *merge sort *algorithm. In order to do this ReCoil creates an array containing the seeds for all reads, sorts this array by numeric representation of the seeds and uses the sorted array to create an anchor for each pair of reads sharing a seed:

This algorithm is illustrated in Figure 1.

1. For each string *S *- a read or its reverse complement - generate all seeds contained in *S*.

2. For some parameter *t *for each read select only the seeds with the *t *topmost numeric representations among all the seeds generated for that read. Output all selected seeds to a file.

3. Sort the file created in step 2 by numeric representation of the seeds.

4. Create an *anchors *file containing pairs of reads sharing a seed. (Below we describe a heuristic to deal with highly repetitive seeds).

5. Sort the anchors file lexicographically using external memory sort.

6. Define the *similarity graph *as an undirected weighted graph with vertices corresponding to the reads and weight of an edge connecting two reads defined as the number of anchors corresponding to the pair of reads. (Note that after the sorting in step 5 the range of anchors corresponding to each pair of reads is contiguous.)

7. Use an external memory MST construction algorithm [11] in order to find the Maximum Spanning Tree in the similarity graph built in the previous step.

In step 2 we use the heuristic introduced in the Minimus Assember [12] in order to limit the number of seeds created by the algorithm.

Note that step 4 of the algorithm is quadratic in the number of reads sharing a seed. Fortunately, if the seeds are long, most of them have relatively low counts. Yet, to restrict the time ReCoil spends on each seed, for the range of reads containing the seed the algorithm first adds the anchors corresponding to the reads that are adjacent in the range, then those with distance of two between them, etc. until some predefined number of anchors per seed were created. In our tests the cut off was set to 80, i.e. we created at most 80 anchors for each seed.

The external memory MST stage has *O*(*Sort n*) I/O complexity [11], where *n *stands for the number of edges in the similarity graph. It is easy to see that all other stages of the algorithm are reductions to either scanning or sorting of their inputs, thus have either *O*(*Scan n*) or *O*(*Sort n*) I/O complexity, where *n *is the size of the input to the stage.

In the following section we explain how we use the similarities between the reads for compression.

### 2.4 Encoding the Dataset

The external memory Kruskal algorithm of step 7 builds an unrooted tree where neither head-to-tail direction on the edges nor any traversal of the spanning tree are computed by the algorithm. Prim's algorithm results in a rooted tree but cannot be implemented efficiently in external memory. One solution to find the directions of edges (*rooting*) of the tree would be to use the external memory Breadth First Search (BFS) algorithm described in [13]. This algorithm computes the BFS order on a tree of *N *vertices in *O*(*Sort n*) I/O operations using a reduction to an external memory algorithm for computing an Eulerian Tour in a graph. Yet while the similarity graph can be very large, its spanning tree can be stored in the RAM of a modern computer for any practical number of reads (vertices). Hence we can use a simple in-memory BFS algorithm for rooting of the spanning tree as it is faster than the external memory BFS for graphs that can fit in RAM.

After the BFS, the algorithm uses an external memory sort to reorder the reads in the order of the BFS traversal of the MST. Let us denote with *S *the BFS-reordered read array and with *p*(*i*) denote the position of a parent of read *S*[*i*] in *S*. By construction, *i < j *⇒ *p*(*i*) ≤ *p*(*j*).

Algorithm 1 scans through the array of BFS edges (*p*(*i*), *i*) and *S *in parallel in order to encode read *S*[*i*] based on read *S*[*p*(*i*)]. The algorithm runs several sequential scans simultaneously. In case of a single disk, *k *simultaneous scans can be implemented using a separate buffer of size *B *for each scan, where *B < M/k*. As a result, the I/O complexity of Algorithm 1 is the same as of the scan: while the scan can use any block size *B < M*, the only difference would be in the multiplicative constant. In the description below, *Load*(*destRAM*, *srcdisk*, *pos*) buffers into a RAM array *destRAM *at most *B *bytes, starting from position *pos *of a disk array *srcdisk*. The value returned by the function is the position of the last element read.

   **Init: **Allocate arrays *Children *and *Parents *of *B *bytes;

   Let *LastParent *= *LastChild *= 0, *i *= 1;

   Write down *S*[0] - the sequence in the root - explicitly;

   **foreach ***BFS edge *(*p*(*i*), *i*) **do**

      **if ***i > LastChild ***then**

      |    *LastChild *= *Load*(*Children*, *S*, *i*);

      **end**

      **if ***p*(*i*) *> LastParent ***then**

      |    *LastParent *= *Load*(*Parents*, *S*, *p*(*i*));

      **end**

      Use the MEM-based encoding of Section 2.5 to encode the sequence *S*[*i*] given *S*[*p*[*i*]] or the reverse complement of *S*[*p*[*i*]], depending on what results in a shorter encoding;

   **end**

   **Finalize: **Use a general purpose compression algorithm, such as *7-zip*, to further improve the compression.

**Algorithm 1: **Encoding of a BFS-ordered sequence *S *of reads.

To illustrate the encoding, for the sequences of Figure 1 we have:

1. Reorder the sequences in the BFS order of the MST, rooted in sequence 1, i.e. to (1, 3, 2): *ACCGTTA, GTTAGCA, CCCTTAG*. Let's label the reordered sequences as 1', 2', 3'.

2. Write the root explicitly: *ACCGTTA*.

3. Encode 2' referring to 1': ReadLen = 7, *MEMs = (SourceID = 1', StartIn-Source = 3, StartInDest = 0, Length = 4)*; *PlainTextLetters *= *GTT*.

4. Encode 3' referring to 2': ReadLen = 7, *MEMs = (SourceID = 2', StartIn-Source = 2, StartInDest = 3, Length = 4)*; *PlainTextLetters *= *CCC*.

To further improve the compression rate, ReCoil uses difference coding to reduce the ranges of the numbers it stores. Like Coil, it uses separate files for storing properties such as read lengths as these values are typically identical and can be compressed well using the final general purpose compression step. The combined I/O complexity of this stage is the same as that of external memory sorting of the reads.

Finally, we show how ReCoil finds the MEM-based encoding of one read relative to another similar read.

### 2.5 Encoding Sequence Similarities

Given two strings *s*_1 _and *s*_2 _we use the following simple algorithm to find all MEMs shared by them:

1. Merge the seeds contained in *s*_1 _and *s*_2 _in one array and sort this array using the seeds' numeric representations as the keys.

2. Scan the sorted array and for each seed shared by both *s*_1 _and *s*_2 _create a tuple (*r*_1_, *r*_2_), where *r*_1 _and *r*_2 _are positions of the seed in *s*_1 _and *s*_2 _respectively.

3. Sort the tuples defined above lexicographically, where the keys are defined as (*r*_1 _*- r*_2_, *r*_1_), i.e. first by diagonal of the match corresponding to the anchor, then by the position of the match in the first read; as a result, the anchors corresponding to each MEM follow sequentially, making it easy to extract the MEMs. A similar sorting strategy was used by Ning et al. [14].

In the next step ReCoil uses the sparse dynamic programming algorithm of Eppstein et al. [15] in order to find the subset of MEMs that results in the optimal compression of *s*_1 _relative to *s*_2_. This algorithm finds the optimal alignment between two sequences under affine gap cost, subject to the restriction that each matching segment is at least *k *nucleotides long. The Gap Open Penalty is defined by the shortest MEM length that is profitable to use for the encoding. For larger values of *k *this algorithm is much faster than the Smith-Waterman [16] algorithm.

Denote by *Gain*(*s*_1_, *s*_2_) the space saved if *s*_2 _is encoded relative to *s*_1 _using the encoding described above. Since for every MEM in the encoding, the space saved is the difference between the memory required to store the location of the MEM versus storing it explicitly, *Gain *function is symmetric and the same amount of space is saved, whether *s*_1 _is encoded relative to *s*_2 _or vice versa.

### 2.6 Decompression

The inputs to the decompressor are several streams produced by the encoder: the read lengths, the list of MEMs shared between the reads and their parents that were used for the encoding, as well as explicitly stored letters of the reads. These streams are read concurrently. Note that the data in the streams is ordered in the BFS order on the encoding spanning tree. As in the case of algorithm 1, the decoding uses several buffers in order to implement multiple sequential accesses to a single disk.

Since the reads are decoded in the BFS order on the encoding tree, in order to decode, we create a stream of the decoded reads maintaining the position of the parent of the currently decoded read. Since this position changes in a non-decreasing order, the decompression step has *O*(*Scan n*) I/O complexity, i.e. *O*(*n/B*) disk accesses, where *B *is the size of the block loaded into memory on each access.

## 3 Results and Discussion

ReCoil was implemented in C++ using the STXXL [17] library of external memory data structures. The *k*-mer size selected was 15 for the similarity graph construction and 10 for the encoding step. We tested ReCoil on both simulated and real datasets. The simulated datasets were generated by making random samples of given length from Human Chromosome 14, adding single-nucleotide errors (insertions, deletions or substitutions) with probability 0.02 and reverse complementing each read with probability 0.5. All our generated datasets were of the same size of 1.8 billion nucleotides.

**Figure 2:**
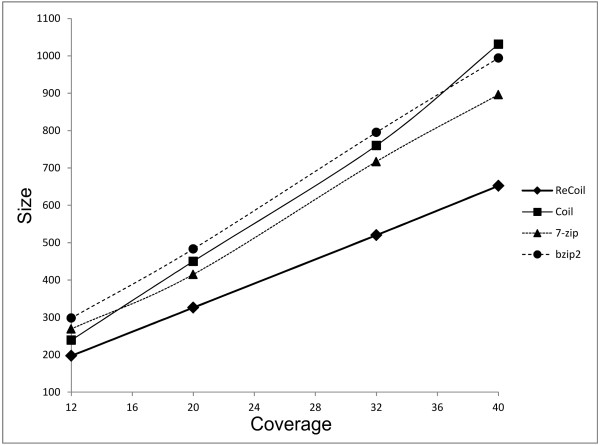
**Comparison of compression with ReCoil, Coil, 7-zip and bzip2 for various coverages**. The simulated datasets were generated by making random samples of length 70 from Human Chromosome 14, adding single-nucleotide errors (insertions, deletions or substitutions) with probability 0.02 and reverse complementing each read with probability 0.5.

The results are summarized in Table 1 and Figure 2. From the results on the synthetic datasets we can see that as the read length increases, the compression rate of ReCoil improves. One explanation for the better compression is that longer reads are more likely to share more and longer MEMs. Also in our tests the run times were decreasing when the length of the synthetic reads was increasing: if the coverage is kept constant, longer reads result in smaller similarity graphs. On the other hand the general purpose algorithms have runtimes and compression rate independent of read length.

**Table 1:** Comparison of compressed file size, runtime and compression ratios of ReCoil to Coil, 7-zip and bzip2.

		ReCoil			Coil			7-zip			bzip2		
**Readlen**	**Megabases**	**Size**	**Time**	**%**	**Size**	**Time**	**%**	**Size**	**Time**	**%**	**Size**	**Time**	**%**

36	6912	1180	840	0.17	NA	NA	NA	1900	300	0.27	2250	45	0.36

70	1800	326	290	0.18	450	650	0.25	412	78	0.23	483	11	0.27

100	1800	278	246	0.15	415	625	0.23	405	76	0.22	481	11	0.27

120	1800	241	198	0.13	387	590	0.21	403	77	0.22	480	11	0.27

File sizes are shown in megabytes and run times in minutes. ReCoil, Coil and 7-zip were given 2 GB of RAM, while bzip2 was run with the compression-level = 9. Both ReCoil and Coil used 7-zip as a post-processing compression step, this step was included in the timings. Decoding time for ReCoil was less than 5 minutes for all datasets, including the 7-zip uncompress step.

There are several reasons why we were able to achieve better compression rate than Coil, 7-zip and bzip2. First, the algorithms we implemented in ReCoil allow us to make use of similarities between the reads located far from each other in the input, as ReCoil does not require splitting of the input into smaller parts. Another reason for better compression of ReCoil than Coil, is the fact that the edges between the reads in our similarity graph reflect better the gain obtained by encoding one read relative to another.

To test ReCoil on a real short read data, we compressed a dataset of 192 million Illumina reads of length 36 downloaded from http://www.ncbi.nlm.nih.gov/sra/SRX001540, which is a part of "Human male HapMap individual NA18507" project http://www.ncbi.nlm.nih.gov/sra/SRP000239. This resulted in a file of size 1.18 GB. *7-zip *compressed the same sequences to size 1.9 GB. *7-zip *was our general purpose compressor of choice in the comparisons since in our experience it resulted in the best compression. Out of 192 million sequences in the dataset, ReCoil stored 4 million sequences explicitly, the rest were stored compressed. We were not able to run Coil on this dataset, while it took about 14 hours for ReCoil to compress this dataset using a 1.6 GHz Celeron with four hard disks and 4 GB of RAM. ReCoil can make use of several disks installed on the machine not only to scale up to large datasets but also to speed up the computations due to higher disk bandwidth. Nevertheless all the algorithms remain efficient if only a single hard disk is present.

We also attempted to compare our algorithm to a publically available reference-based compression algorithm MZip [8]. Unfortunately it could not scale to the size of our datasets as just its preprocessing step, converting the results of a BWA mapping program to its internal format took more than 90 minutes on a set of 3.5 million reads of length 36, not counting the BWA alignment step itself, and we were unable to run the pipeline to completion.

## 4 Limitations and Future Work

Our goal in this work was to design an algorithm that maximizes compression ratio. Yet the ability to retrieve a sequence without full decompression will improve applicability of the algorithm. One simple way to accomplish this would be to store the sequences at some levels of the spanning tree explicitly. Then in order to decode a sequence of a node one must go up in the tree until reaching a level of not encoded nodes.

One limitation of ReCoil in practice is that it only deals with the sequencing data, while compressing the metadata such as quality values is as important. Another limitation of ReCoil's approach is that in order to limit the number of anchors corresponding to repeats and to reduce the number of seeds created we had to use various heuristics that compromise how well the weights of the edges of the similarity graph reflect the savings from encoding one read relative to another. Ferragina et al. [18] describe an algorithm for computing the BWT transform in external memory. While we saw in our testing that BWT-based *bzip2 *provided significantly worse compression than ReCoil, one direction for future work would be to use a BWT-based approach in order to find the MEMs and build the similarity graph more efficiently.

## 5 Competing interests

The author declares that he has no competing interests.

## 6 Authors' contributions

VY conceived of the study, implemented the algorithms and tested them.
